# Heavy Metals in Acrylic Color Paints Intended for the School Children Use: A Potential Threat to the Children of Early Age

**DOI:** 10.3390/molecules26082375

**Published:** 2021-04-19

**Authors:** Mohammad Rizwan Khan, Naushad Ahmad, Mohamed Ouladsmane, Mohammad Azam

**Affiliations:** Department of Chemistry, College of Science, King Saud University, Riyadh 11451, Saudi Arabia; anaushad@ksu.edu.sa (N.A.); mouladsmane@ksu.edu.sa (M.O.); mhashim@ksu.edu.sa (M.A.)

**Keywords:** heavy metals, acrylic color paints, school children, cancer, iCAPQ ICP-MS

## Abstract

Heavy metals are the harmful elements, regarded as carcinogens. Nevertheless, owing to their physical and chemical properties, they are still used in the production of several commercial products. Utilization of such products increases the chance for the exposure of heavy metals, some of them are categorized as probable human carcinogens (Group 1) by the International Agency for Research on Cancer. Exposure of heavy metals to school children at early age can result severe life time health issues and high chance of emerging cancer. Thus, we have performed study relating to the presence of heavy metals in acrylic color paints commonly used by the school children. Acrylic paints of different colors were assayed for seven potential heavy metals manganese (Mn), cobalt (Co), nickel (Ni), zinc (Zn), arsenic (As), cadmium (Cd) and lead (Pb) using microwave digestion and iCAPQ inductively coupled plasma mass spectrometry (ICP-MS) system. The optimized method including paints digestion reagents nitric acid (HNO_3_, 65%, 5 mL) and hydrofluoric acid (HF, 40%, 2 mL) have offered excellent method performance with recovery values ranged between 99.33% and 105.67%. The elements were identified in all of the analyzed samples with concentrations ranged from 0.05 to 372.59 µg/g. Cd constitutes the lower percentage (0.05%), whereas Zn constitutes high ratio contribution which was tremendously high (68.33%). Besides, the paints contamination was also color specific, with considerably total heavy metal concentrations found in brunt umber (526.57 µg/g) while scarlet color (12.62 µg/g) contained lower amounts. The outcomes of our investigation highlight the necessity for guidelines addressing the heavy metals in acrylic color paints intended for the school children usage.

## 1. Introduction

Environmental health risk by means of colorants based on heavy metals is a persistent menace to humans, particularly children of early age. Heavy metals are frequently used as colorants in childrens’ toys, cosmetics and face paints [1,2,3,4,5]. Owing to their great degree of toxicity, the heavy metals including lead (Pb), arsenic (As), chromium (Cr), mercury (Hg) and cadmium (Cd) are categorized among the priority hazardous metals of human health importance [6,7]. The deadliness of metals depends on numerous aspects, for instance the dose, exposure route and type of compound, in addition to the individual’s heredity, gender, age and dietary conditions [8]. The capability of heavy metals to enter into the systemic circulation system through dermal absorption and percolation may cause their agglomeration and subsequent adverse influences [9,10,11]. They are identified as universal toxicants recognized to cause several kinds of organ damage, even after exposure to minor amounts [11]. The main routes of heavy metal absorption are inhalation and ingestion via paints and foods [12]. Relating to mineralization, the human body has mild acid and base conditions and not the whole amount of the heavy metals in paint is bioavailable [13,14]. The bioavailability of heavy metals is regulated by several physical, chemical and biological factors for example adsorption, phase association, temperature, thermodynamic equilibrium speciation, lipid solubility, kinetics complexation, biochemical/physiological adaptation and species characteristics [13,14]. Heavy metals have biochemical and physiological functions, and are essential components of several important enzymes which play significant roles in many redox reactions [13]. Regarding the exposure of humans to Pb, it leads to brain damage and anemia [15,16,17]. The bones, blood and soft tissues are the main human body organs that accumulate Pb [18], and deposits in teeth and bones account for the more severe health effects, especially in children [19]. The main source of Pb toxicity is its interference with different enzymes present in the human body which generate reactive radicals that harm the cell structures comprising cell membranes and DNA [20]. Exposure to As plays an important role in the development of vascular endothelial deformity in the human body, leading to a diminution in the bioavailability and generation of nitric oxide. Besides, prolonged As exposure causes high oxidative stress, which leads to cardiovascular and central nervous system dysfunction [21] and skin cancer [22]. Cd exposure produces skin tumors and toxicity to the kidneys [23], Cd is readily retained in the proximal tubular cells of the human body. It accumulates over the course of a lifetime which results in bone demineralization either via bone damage or as a result of renal dysfunction [24]. Mn exposure leads to a neurodegenerative illness that causes dopaminergic neuronal death [25,26]. Mn accumulates mainly in the bones, human plasma and the brain [27,28,29]. Mn is measureable in the cerebrospinal fluid before it can be identified in the brain parenchyma, signifying that it is carried by means of the choroid plexus [30]. The brain is considered as the main target organ of Mn toxicity [29]. Zn exposure has adverse effects on the gastrointestinal system, reproduction, hematemesis, and it is a contact allergen and causes renal injuries [31,32]. Zn is mainly absorbed in the second part of the small intestine (jejunum) in humans. Absorption is predominantly assisted by means of metallothionein protein in the intestinal villi of the cell of the intestinal lining. Zn is then bound to the metallothionein which controls the concentrations of several other metals. To some extent, homeostasis is controlled by the metallothionein-zinc complex excretion through feces and bile [32]. Ni exposure has adverse effects as a contact allergen [33]. Higher doses may be carcinogenic and even lethal, and create an occupational threat [32,34]. Ni compounds are categorized as human carcinogens [34,35] based on greater respiratory cancer incidence detected in Ni epidemiology investigations [35].

The maximum health threat assessments link human exposure to heavy metals in other sources such as water, air, newspapers, wall paints, furniture dust, toys and soil [1,2,3,4,5,36,37,38,39,40,41]. Experimental and epidemiological investigations have revealed a link between heavy metal exposure and cancer occurrence in animals and human beings [36,42,43]. The International Agency for Research on Cancer (IARC) and the World Health Organization have regulated and categorized them as probable human carcinogens [36,42,44,45]. The German Federal Agency for Consumer Protection and Food Safety (GFACPFS) has issued the strictest guidelines relating to the presence of some heavy metals (Pb, As and Cd) in cosmetic products, where the levels must be 2, 0.5 and 0.1 μg/g, correspondingly [46].

Recently, Wang et al., have identified the heavy metals in face paints, finding average amounts of Pb (16.2 μg/g), As (1.8 μg/g), Cd (0.6 μg/g), Cr (23.1 μg/g), Co (4.4 μg/g), Cu (610 μg/g), Zn (10415 μg/g) and Ni (7.6 μg/g), and as a minimum four of the eight heavy metals were identified in all of the analyzed samples [2]. The overall carcinogenic threat constituted by the metals in analyzed face paints was 0.01–0.96% [2]. Usually, paint producers use heavy metals deliberately in paint preparations for many causes such as color development, to produce a premier class of paints and protecting surfaces from deterioration and corrosion [41,47]. With the growing trend of the economy, population and industry growth worldwide, paints containing heavy metals are on the markets in all nations, particularly those where no strict regulations are available to control the levels of heavy metals [8,48], and most of the people are less familiar with their threats. Overall the heavy metal exposure constitutes a high health threat to all human beings, and among them the children are assumed to be at greater risk [49]. Children are at high risk of gastrointestinal heavy metal absorption and this results in neurological and behavioral illnesses, for example mental retardation and poor memory [50,51].

In order to reduce the adverse human health influences caused by heavy metal-based paints [2,48,52], the monitoring of heavy metals in acrylic paints is of high importance.

Acrylic paint is made of a small amount of pigments (natural, synthetic, inorganic, and organic) and an acrylic polymer emulsion (plastic acrylic resin suspended in water). As the water vaporizes the resin particles fuse together resulting in a sturdy paint [53]. Based on the viscosity/thickness of the acrylic paint, various types acrylic paints are commercially available such as heavy body acrylic, soft body acrylic, fluid acrylic, acrylic markers, acrylic inks and acrylic gouache [54]. Acrylic paints are one of most multifaceted media. Acrylic paints are water-soluble when wet and water insoluble when dry and they transform into flexible colors. Acrylic paints are water resistant, providing a sturdy surface to which successive paint layers can be applied without affecting the underlying coatings. They can be washed by means of water [54]. Effective actions targeting obstructing exposure to heavy metals from paints intended particularly for childrens’ use will help to ensure their health and also promote safety for individuals of all ages, and therefore, have a considerable impact on the reduction of diseases and deaths caused by lethal chemicals present in food, water, soil and air pollution.

## 2. Results and Discussion 

### 2.1. Optimization of Microwave Digestion Method

Following a very simple sample digestion step, an iCAPQ ICP-MS system is routinely proficient of determining minor and major elemental levels in highly matrixed samples. Because of the high sensitivity and robustness of iCAPQ ICP-MS, the simultaneous estimation of all compounds of interest in an extensive range of highly complexed samples can be determined proficiently, interference-free and speedily [55,56].

**Figure 1:**
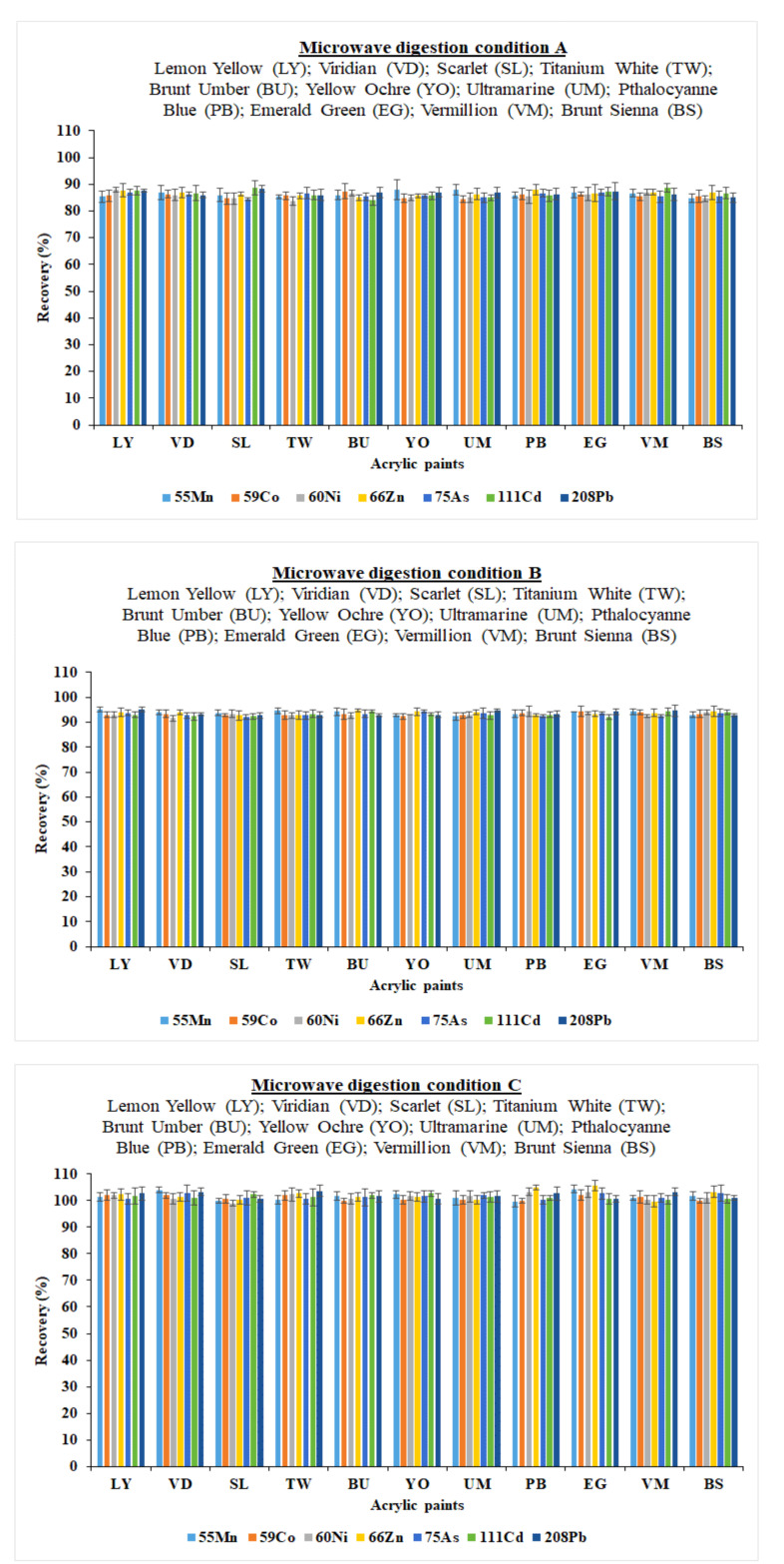
Recovery values with standard deviation error bar achieved from the three optimized microwave digestion methods.

Prior to the analysis, initially, digesting reagents such as HNO_3_ (65%) and HF (40%) were chosen for the sample digestion purposes. These reagents were chosen because of highly matrix nature of the paint samples. The three microwave digestion procedures (Table 1) were chosen by varying the main conditions, for instance temperature (°C), hold time (min) and power (%). Approximately 0.1 g dried paint samples was placed in the pressure vessels of the microwave digestion system followed by the addition of the digesting reagents HNO_3_ (65%, 5 mL) and HF (40%, 2 mL). The sample was left for 10 min at room temperature for pre-digestion and then placed in microwave system for pressure digestion. In Method A, at first, the pressure digestion was performed by keeping the pressure at a constant value (90 bar) and varying the temperature (160-180-40 °C), hold time (5-30-10 min) and power (60-70-0%). The obtained recovery values were between 84.33% and 88.67%. In Method B, the pressure was held constant (90 bar) and the temperature (170-190-50 °C), hold time (8-40-15 min) and power (70-80-0%) was varied. The recovery values were slightly increased, and ranged from 92.00% and 95.37%. In Method C, the pressure was kept constant (90 bar) and the temperature (180-200-60 °C), hold time (10-50-20 min) and power (80-100-0%) was varied. In this case, excellent recovery values were achieved, ranging from 99.67% to 105.67%. The recovery values with standard deviation error bar achieved from the three optimized microwave digestion methods are presented in Figure 1.

The samples have all been analyzed by the iCAPQ ICP-MS system (Table 2), and the heavy metal (Mn, Co, Ni, Zn, As, Cd and Pb) concentrations determined. To check the accuracy of the method, the samples were fortified with the known heavy metal concentrations and analyzed by same method as described above.

Wang et al. have studied the identification of heavy metals (As, Cd, Pb, Co, Cr, Cu, Ni, and Zn) in face paints, obtaining recovery values between 87–110% [2]. The recovery values were thus in good agreement with those obtained in the present work. Nonetheless, the digestion procedure was slightly different, comprising mixed concentrated acids (HNO_3_, 7 mL, H_2_O_2_, 2 mL and HF 1 mL), microwave-accelerated digestion and an ICP-MS system [2]. In another similar study, Silvia et al. obtained 80–114% recovery rates for Al, As, Ba, Cd, Cr, Cu, Mn, Ni, Pb, Sn, Sr, Ti and Zn in paint samples using a new wet digestion procedure (HCl, 5 mL and HF, 1 mL) of HF and inductively coupled plasma optical emission spectrometry (ICP OES) [52]. These values were also found in good agreement with those obtained in the current study.

### 2.2. Performance of the Method

**Figure 2:**
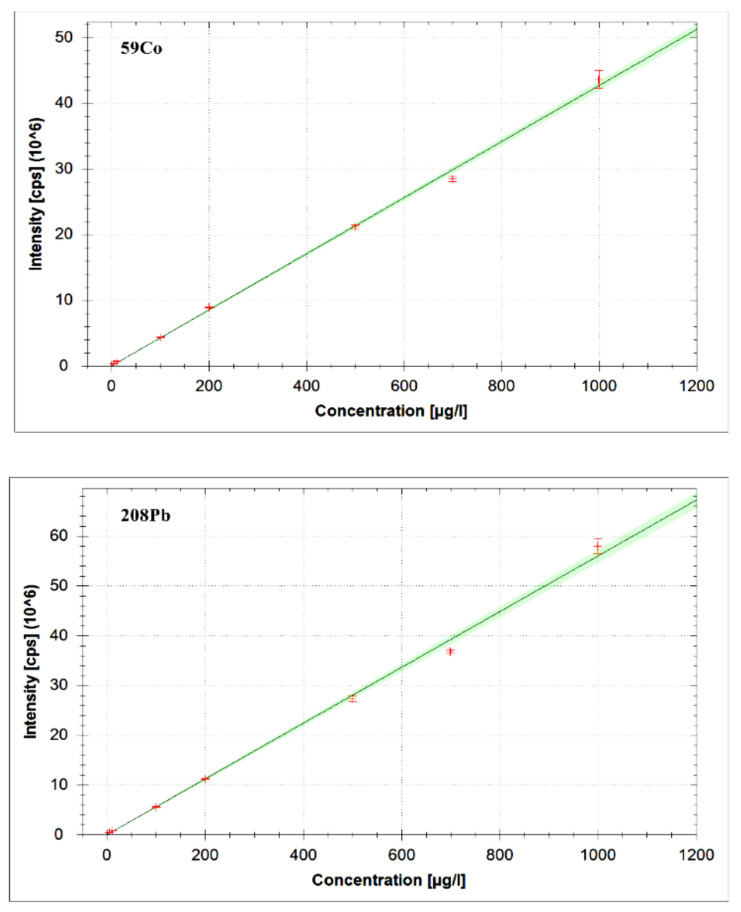
Calibration curves of some of the heavy metals (Co and Pb) obtained using iCAPQ ICP-MS system.

The method performance was determined in terms of coefficient of determination (CoD, R^2^), limit of detection (signal-to-noise ratio 3:1); LOQ, limit of quantification (signal-to-noise ratio 10:1), precision and accuracy. The CoD was determined by constructing calibration curves for the heavy metals (Mn, Co, Ni, Zn, As, Cd and Pb) in the concentration range from 5–1000 µg/L. The CoD (R^2^) of the method was found to be between 0.9784–0.9984. As an example, the calibration curves of some of the heavy metals (Co and Pb) obtained using the iCAPQ ICP-MS system are presented in Figure 2.

The outcomes show that the method offers linearity from lower (5 µg/L) to higher (1000 µg/L) concentrations of the heavy metals (Table 3). LOD and LOQ values were determined from the individual calibration equations applying method based on 3 × standard deviation of the response/slope. LOD and LOQ values were found in the range of 0.0033 to 0.6055 µg/L and 0.0102 to 1.8169 µg/L, correspondingly. Excellent LOD LOQ values were identified and suggest the method could applicable to determine these elements in highly matrixed samples. The achieved values are displayed in Table 3. The precision (repeatability and reproducibility) of the method was determined by analyzing a mixed solution of heavy metals at lower (5 µg/L) and medium (500 µg/L) concentrations. The repeatability was tested by five injections of the both low and medium standard solutions three times in a day. The reproducibility was verified by five injections of the both low and medium standard solutions for three consecutive days. The values were estimated in terms of relative standard deviation (RSD%), giving results lower than <4% for repeatability and <7% for reproducibility at both the levels. The obtained values are similar to those obtained in an earlier study [52]. Recovery values of the studied elements were between 99.67% to 105.67%, achieved using element addition and the found concentrations.

### 2.3. Application

**Figure 3:**
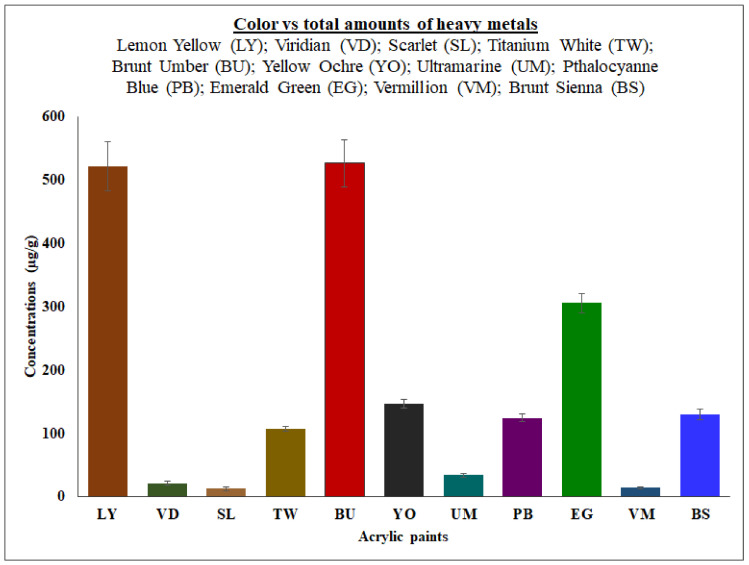
Acrylic color paints vs variation of heavy metal concentrations.

The optimized method was applied for the analysis of heavy metals (Mn, Co, Ni, Zn, As, Cd and Pb) in acrylic color paints intended for school childrens’ use. The study involved a sample comprising eleven different colors, including lemon yellow (LY), viridian (VD), scarlet (SL), titanium white (TW), brunt umber (BU), yellow ochre (YO), ultramarine (UM), pthalocyanne blue (PB), emerald green (EG), vermilion (VM) and brunt sienna (BS). Based on our survey, acrylic color paints are highly used color paints in schools at the primary level. The measured amounts of Mn, Co, Ni, Zn, As, Cd and Pb are presented in Table 4. The outcomes show that the studied elements (Mn, Co, Ni, Zn, As, Cd and Pb) were found in all the samples in amounts ranging from 0.05 to 372.59 µg/g. Among them, the Cd had the lower percentage (0.05%), whereas Zn was the most abundant metal present, with a tremendously high amount (68.33%). In accordance with USEPA and the Agency for Toxic Substances and Disease Registry (ATSDR) [10] and GFACPFS [46], the elements As, Cd and Pb are the key ones in the list of hazardous substances lethal to human health, and consumption of these elements, even at small amounts, is extremely toxic [10,46,57]. As for As, the analyzed samples (SL, EG and VM) contained low concentrations of As, ranging from 0.17 to 0.92 µg/g, however other samples (LY, VD, TW, BU, YO, UM, PB and BS) contained higher concentrations, ranging from 1.23 to 8.14 µg/g. The Cd levels were found to be at lower levels in all of the analyzed samples, ranging from 0.05 to 0.19 µg/g. Concerning the levels of Pb, samples VD, SL, YO, PB, VM and BS contained 0.40 to 0.74 µg/g, while samples LY, TW, BU, UM and EG contained 1.34 to 5.03 µg/g. The amounts of heavy metals measured in this study were similar or lower amounts with those obtained previously in in childrens’ play paints [4] and face paints [2]. However, the heavy metals levels were found beyond the values of As (0.5 μg/g), Cd (0.1 μg/g) and Pb (2 μg/g) recommended by the GFACPFS in cosmetic products [46]. The global carcinogenicity risk constituted by the metals in analyzed face paints were 0.01-0.96% [2]. Besides, paint contamination is also color specific, with considerable total heavy metal concentrations found in BU (526.57 µg/g) while SL (12.62 µg/g) contained lower amounts. The color vs variation of heavy metals levels has been presented in Figure 3.

The amounts of heavy metals in the diverse color paints varied greatly. Based on the outcomes obtained in this study, school children of early age may unintentionally be expose to higher amounts of some carcinogenic heavy metals, thus leading to a high chance of emerging cancer disease. Metal exposure poses great health threats to the broader population [58] however children are assumed to be at greater risk than grown persosn because of the increased gastrointestinal absorption of metals in children [58]. Metals have been linked to many diseases in children such as mental retardation with neurological illnesses and poor memory [59]. Many investigations to establish the total amounts of metals in paints have been presented global. The outcomes show that a majority paints had a confirmed presence of different kinds of heavy metals. Lately, an epidemiological assessment was carried out in which hundreds of school children were surveyed for the presence of heavy metals. The study showed that nearly 42% of all children were identified to have detectable blood heavy metal concentrations, especially of Pb. The values were found beyond the tolerable blood concentrations of 10 μg/dL [58].

## 3. Materials and Methods

### 3.1. Reagents and Chemicals

For sample preparation, nitric acid (HNO_3_, 65%) and hydrofluoric acid (HF, 40%) were purchased from Loba Chemie (Mumbai, India). Ultrapure water (Milli-Q) was used to prepare all the solutions, and was acquired from a Milli-Q water purification system (Millipore, Bedford, MA, USA). Conical centrifuge tubes (50 mL) were obtained from Thermo Scientific, Rochester, NY, USA. Aluminum foil was purchased from Sanita, (Riyadh, Saudi Arabia). The high-performance oven used to dry the paint samples was obtained from Shel Lab (Cornelius, OR, USA). Glassware was submerged in 10% HNO_3_ for twenty-four hours and washed several times with Milli-Q water prior to use for sampling purposes. Standard solutions were prepared after sequential dilutions from certified reference material (ICP/MS calibration standard, 10 µg/mL) stock solutions of (Mn), cobalt (Co), nickel (Ni), zinc (Zn), arsenic (As), cadmium (Cd) and lead (Pb) (Ultra Scientific, North Kingstown, RI, USA). For element analysis, the calibration curve solution was prepared in the range between 5 µg/L and 1000 µg/L.

### 3.2. Sample Collection

A pack of eleven water-based acrylic color paints of an international brand was purchased from a local stationary shop in Riyadh, Saudi Arabia. The paints were specifically intended for the use of school children. Paint samples were of different colors, for instance lemon yellow (LY), viridian (VD), scarlet (SL), titanium white (TW), brunt umber (BU), yellow ochre (YO), ultramarine (UM), pthalocyanin blue (PB), emerald green (EG), vermilion (VM) and brunt sienna (BS). Each paint tube contained a volume of 12 mL, and their expiry dates were labeled.

### 3.3. Sample Analysis

**Figure 4:**
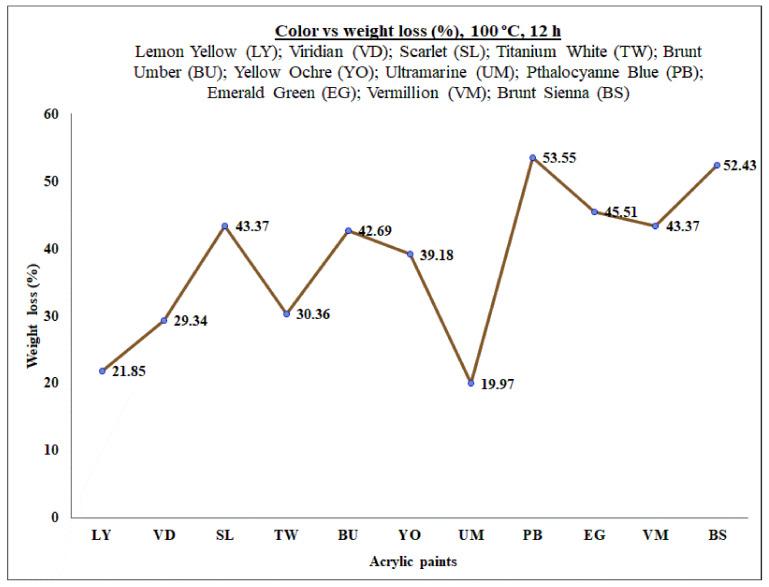
Color vs. weight loss of the acrylic paint samples.

Each paint sample (~2 g) were weighed on an aluminum foil using a precise analytical balance (Rawag^®^, Wagi Elektroniczne, Kopernika, Poland). To eliminate the water and volatile organic substances, the paint samples were dried in an oven at 100 °C for 12 h. The weight loss was calculated after drying the samples. The color vs weight loss data of the acrylic paint samples is presented in Figure 4.

Subsequently, the samples were homogenized manually using a laboratory mortar. Following the sample digestion, a known amount (~0.1 g) of homogenized sample was directly weighed into weighing cups made of modified polytetrafluorethylene (TFM-PTFE). Then, the sample were placed in pressure vessels (TOPwave, PM 60, Analytik Jena AG, GmbH, Munich, Germany) containing an aluminum rupture disc, and a coupling cap made of TFM-PTFE. The pressure vessel volume was 60 mL with a maximum pressure rating of 60 bar, temperature rating of 210 °C (continuous operation), sample weight < 500 mg and minimum fill volume (acid) > 7 mL. The digestion reagents HNO_3_ (65%, 5 mL) and HF (40%, 2 mL) were added to the pressure vessel containing the paint sample. In order to pre-digestion the samples, they were left for 10 min at room temperature and then placed in a microwave system for pressure digestion (TOPwave, Analytik Jena AG, GmbH). The optimized conditions are presented in Table 1 (Conditions C). The total digestion time was 1 h 20 min. After the digestion process, the samples were allowed to stabilize and transferred to conical centrifuge tubes (50 mL). Then, the sample was diluted with Milli-Q water (free from heavy metals) to a volume of 25 mL, and left for 10 min for the matrices to settle down. After that, the samples were analyzed by the iCAPQ ICP-MS system which is known to be an excellent system for multi-elemental identification in highly complex samples [55,56]. Since paint sample certified materials were not available, the method accuracy was determined by means of addition/recovery assessments. To carry out this assessment, a determined amount of heavy metal was added to each sample prior to the digestion process and iCAPQ ICP-MS analysis. The achieved values were compared with added and found concentrations. The samples and blanks were analyzed for 30 s.

### 3.4. iCAPQ ICP-MS

Heavy metals, for instance Mn, Co, Ni, Zn, As, Cd and Pb in water-based acrylic paints were identified using an iCAPQ ICP-MS system (Thermo Scientific™, Bremen, Germany). The system comprises a nebulizer, Peltier-cooled spray chamber, peristaltic pump, torch with the injector, interface, mass analyzer and dual mode secondary electron multiplier detector. The main iCAPQ ICP-MS operating conditions were: forward power (1548.6 W), interface temperature (37.9 °C), cool gas flow (13.881 L/min), auxiliary gas flow (0.7977 L/min), nebulizer gas flow (0.9692 L/min), spray chamber temperature (−20 °C). The system operation mode was standard. The detailed iCAPQ ICP-MS operating conditions have been listed in Table 2. The system was monitored using Qtegra™ Intelligent Scientific data solution software (Thermo Scientific™).

The quantification of each element was carried out using calibration curves from a mixture of standard solutions comprising the heavy metals (Mn, Co, Ni, Zn, As, Cd and Pb). The limits of detection (LOD, signal-to-noise, 3:1) and limit of quantification (LOQ, signal-to-noise, 3:10) of the heavy metals were estimated by the three-fold deviation from three replicates (*n* = 3). The recovery values were achieved by fortifying the paint samples with a known concentration of heavy metals. The heavy metals recovery rates were estimated as the ratio of the variance of a determined metal amount between the fortified and non-fortified paint samples to the fortified metal concentration. Statistical analysis was performed using two-way analysis of variance (ANOVA). In the current study, some of the variance (instrument interferences) relating to the identification has not been tested and this parameter will be considered in a future study.

## 4. Conclusions

The optimized method offered excellent method performance, for instance CoD, LOD, LOQ and precision with recovery values of 99.33% and 105.67%. Based on our results, acrylic color paints commonly used by school children were highly contaminated with the heavy metals Mn, Co, Ni, Zn, As, Cd and Pb, with the maximum metal amounts identified in samples being 372.59 µg/g. Most of the analyzed heavy metals levels were found beyond the limits recommended by the USEPA, IARC, and GFACPFS. In addition, paint contamination was also color specific, with considerably total heavy metal concentrations found in LY and BU (>500 µg/g), EG (>300 µg/g), TW, YO, PB, VM and BS (>100 µg/g), and VD, SL, UM, and VM (>10 µg/g). The amounts of heavy metal in the diverse color paints were highly variable (Figure 3). The information from in this investigation offers useful evidence about the levels of certain heavy metals present in school children’s acrylic color paints and associated to the potential threat of exposure to these hazardous elements which may lead to the emergence of cancer diseases.

## Figures and Tables

**Table 1:** Microwave digestion operating conditions.

**Condition A**					
Step	Temperature (°C)	Pressure (bar)	Ramp time (min)	Hold time (min)	Power (%)
1	160	90	5	5	60
2	180	90	5	30	70
3	40	90	1	10	0
**Condition B**					
Step	Temperature (°C)	Pressure (bar)	Ramp time (min)	Hold time (min)	Power (%)
1	170	90	5	8	70
2	190	90	5	40	80
3	50	90	1	15	0
**Condition C**					
Step	Temperature (°C)	Pressure (bar)	Ramp time (min)	Hold time (min)	Power (%)
1	180	90	5	10	80
2	200	90	5	50	100
3	60	90	1	20	0

**Table 2:** iCAP Q ICP-MS operating parameters.

Parameter	Value
Forward power	1548.6 W
Interface temperature	37.9 °C
Cooling water flow	3.46 L/min
Cool gas flow	13.881 L/min
Auxiliary gas flow	0.7977 L/min
Nebulizer gas flow	0.9692 L/min
Pirani pressure	1.581 × 10^0^ mbar
Penning pressure	2.943 × 10^−7^ mbar
Mode of operation	STD
Peristaltic pump rate	40 rpm
Nebulizer	Glass concentric type
Spray chamber temperature	−20 °C
Injector	Quartz, 2.5 mm ID
Torch	Two concentric quartz tubes
Sample tubing	0.508 mm ID, STD
Drain tubing	1.29 mm ID, STD
Star-end mass	4.60–245 u
Dwell time	0.01 s
Number of replicates	3
Sample uptake	40 s
Wash time	30 s
Lens tune	Auto tune

STD, Standard; rpm, revolutions per minute; ID, internal diameter.

**Table 3:** Results of regression equations, CoD, BEC, LOD and LOQ obtained by iCAP Q ICP-MS.

Analyte	Conc. Range (µg/L)	Regression Equation	CoD (R^2^)	BEC (µg/L)	LOD (µg/L)	LOQ (µg/L)
55Mn	5–1000	Y = 61710.6613x + 19411.8513	0.9988	0.315	0.0248	0.0747
59Co	5–1000	Y = 42657.9176x + 906.7047	0.9982	0.021	0.0033	0.0102
60Ni	5–1000	Y = 8830.1596x + 3527.1885	0.9814	0.399	0.0746	0.2242
66Zn	5–1000	Y = 5123.8806x + 12026.0813	0.9784	4.421	0.3873	1.1623
75As	5–1000	Y = 7302.5973x + 73.3339	0.9834	2.347	0.6055	1.8169
111Cd	5–1000	Y = 7302.5973x + 73.3339	0.9847	0.010	0.0063	0.0193
208Pb	5–1000	Y = 55981.0361x + 14618.7154	0.9968	0.261	0.0093	0.0283

Conc., Concentration; CoD, coefficient of determination; BEC, background equivalent concentration; LOD, limit of detection (signal-to-noise ratio 3:1); LOQ, limit of quantification (signal-to-noise ratio 10:1).

**Table 4:** Amounts of heavy metals found in acrylic color paints commonly used by the school children.

Sample	55Mn (µg/g ± sd)	59Co (µg/g ± sd)	60Ni (µg/g ± sd)	66Zn (µg/g ± sd)	75As (µg/g ± sd)	111Cd (µg/g ± sd)	208Pb (µg/g ± sd)
LY	4.34 ± 0.09	2.66 ± 0.92	1.23 ± 0.11	507.87 ± 8.39	3.15 ± 0.05	0.19 ± 0.03	1.81 ± 0.16
VD	11.87 ± 0.17	1.57 ± 0.33	1.72 ± 0.06	4.27 ± 0.24	1.23 ± 0.07	0.05 ± 0.03	0.74 ± 0.06
SL	3.60 ± 0.19	1.65 ± 0.74	1.04 ± 0.10	4.94 ± 0.14	0.92 ± 0.13	0.05 ± 0.03	0.43 ± 0.06
TW	1.42 ± 0.07	1.04 ± 0.75	0.70 ± 0.06	99.66 ± 3.18	2.23 ± 0.17	0.09 ± 0.01	2.27 ± 0.11
BU	372.59 ± 3.84	14.09 ± 2.25	18.54 ± 0.38	116.49 ± 1.36	3.45 ± 0.19	0.08 ± 0.01	1.34 ± 0.11
YO	12.69 ± 0.64	4.18 ± 0.96	3.45 ± 0.02	121.35 ± 1.21	3.94 ± 0.18	0.06 ± 0.02	0.64 ± 0.01
UM	13.02 ± 0.56	1.36 ± 0.23	2.30 ± 0.11	10.84 ± 0.45	1.43 ± 0.20	0.16 ± 0.01	5.03 ± 0.39
PB	15.18 ± 0.36	5.18 ± 1.01	12.64 ± 0.80	82.81 ± 3.28	8.10 ± 0.19	0.09 ± 0.02	0.62 ± 0.04
EG	11.18 ± 0.41	2.18 ± 0.45	1.69 ± 0.08	288.13 ± 9.69	0.82 ± 0.16	0.08 ± 0.02	1.42 ± 0.05
VM	3.46 ± 0.15	2.38 ± 0.09	1.56 ± 0.20	5.99 ± 0.41	0.17 ± 0.07	0.07 ± 0.02	0.40 ± 0.03
BS	15.82 ± 0.66	6.17 ± 1.10	13.24 ± 0.62	86.34 ± 3.75	8.14 ± 0.58	0.09 ± 0.02	0.60 ± 0.02

sd, standard deviation (*n* = 3).

## References

[1] Cui X.-Y., Li S.-W., Zhang S.-J., Fan Y.-Y., Ma L.Q. (2015). Toxic metals in children’s toys and jewelry: Coupling bioaccessibility with risk assessment. Environ. Pollut..

[2] Wang B., Su Y., Tian L., Peng S., Ji R. (2020). Heavy metals in face paints: Assessment of the health risks to Chinese opera actors. Sci. Total Environ..

[3] Ullah H., Noreen S., Rehman A., Waseem A., Zubair S., Adnan M., Ahmad I. (2017). Comparative study of heavy metals content in cosmetic products of different countries marketed in Khyber Pakhtunkhwa, Pakistan. Arab. J. Chem..

[4] Rebelo A., Pinto E., Silva M.V., Almeida A.A. (2015). Chemical safety of children’s play paints: Focus on selected heavy metals. Microchem. J..

[5] Saadatzadeh A., Afzalan S., Zadehdabagh R., Tishezan L., Najafi N., Seyedtabib M., Noori S.M.A. (2019). Determination of heavy metals (lead, cadmium, arsenic, and mercury) in authorized and unauthorized cosmetics. Cutan. Ocul. Toxicol..

[6] Achparaki M., Thessalonikeos E., Tsoukali H., Mastrogianni O., Zaggelidou E., Chatzinikolaou F., Vasilliades N., Raikos N. (2012). Heavy metals toxicity. Aristotle Univ. Med J..

[7] Paschal D., Burt V., Caudill S., Gunter E., Pirkle J., Sampson E., Miller D., Jackson R. (2000). Exposure of the US population aged 6 years and older to cadmium: 1988–1994. Arch. Environ. Contam. Toxicol..

[8] da Rocha Silva J.P., Salles F.J., Leroux I.N., da Silva Ferreira A.P.S., da Silva A.S., Assunção N.A., Nardocci A.C., Sato A.P.S., Barbosa F., Cardoso M.R.A. (2018). High blood lead levels are associated with lead concentrations in households and day care centers attended by Brazilian preschool children. Environ. Pollut..

[9] Morais S., Costa F.G., Pereira M.d.L. (2012). Heavy metals and human health. Environ. Health Emerg. Issues Pract..

[10] Jose A., Ray J.G. (2018). Toxic heavy metals in human blood in relation to certain food and environmental samples in Kerala, South India. Environ. Sci. Pollut. Res..

[11] Sah D., Verma P.K., Kumari K.M., Lakhani A. (2017). Chemical partitioning of fine particle-bound As, Cd, Cr, Ni, Co, Pb and assessment of associated cancer risk due to inhalation, ingestion and dermal exposure. Inhal. Toxicol..

[12] Patrick L. (2006). Lead Toxicity, a review of the literature. Part I: Exposure, Evaluation, and treatment. Altern. Med. Rev..

[13] Hamelink J., Landrum P.F., Bergman H., Benson W.H. (1994). Bioavailability: Physical, Chemical, and Biological Interactions.

[14] World Health Organization (1997). Trace Elements in Human Nutrition and Health.

[15] Mason L.H., Harp J.P., Han D.Y. (2014). Pb neurotoxicity: Neuropsychological effects of lead toxicity. Biomed Res. Int..

[16] Bartrem C., Tirima S., von Lindern I., von Braun M., Worrell M.C., Mohammad Anka S., Abdullahi A., Moller G. (2014). Unknown risk: Co-exposure to lead and other heavy metals among children living in small-scale mining communities in Zamfara State, Nigeria. Int. J. Environ. Health Res..

[17] Ramírez Ortega D., González Esquivel D.F., Blanco Ayala T., Pineda B., Gómez Manzo S., Marcial Quino J., Carrillo Mora P., Pérez de la Cruz V. (2021). Cognitive Impairment Induced by Lead Exposure during Lifespan: Mechanisms of Lead Neurotoxicity. Toxics.

[18] Karri S.K., Saper R.B., Kales S.N. (2008). Lead encephalopathy due to traditional medicines. Curr. Drug Saf..

[19] Barbosa F., Tanus-Santos J.E., Gerlach R.F., Parsons P.J. (2005). A critical review of biomarkers used for monitoring human exposure to lead: Advantages, limitations, and future needs. Environ. Health Perspect..

[20] Flora S., Mittal M., Mehta A. (2008). Heavy metal induced oxidative stress & its possible reversal by chelation therapy. Indian J. Med Res..

[21] Konduri G.G., Bakhutashvili I., Eis A., Gauthier K.M. (2009). Impaired voltage gated potassium channel responses in a fetal lamb model of persistent pulmonary hypertension of the newborn. Pediatric Res..

[22] Huang H.-W., Lee C.-H., Yu H.-S. (2019). Arsenic-induced carcinogenesis and immune dysregulation. Int. J. Environ. Res. Public Health.

[23] Hsueh Y.-M., Lin Y.-C., Huang Y.-L., Shiue H.-S., Pu Y.-S., Huang C.-Y., Chung C.-J. (2021). Effect of plasma selenium, red blood cell cadmium, total urinary arsenic levels, and eGFR on renal cell carcinoma. Sci. Total Environ..

[24] Bernard A. (2008). Cadmium & its adverse effects on human health. Indian J. Med Res..

[25] Emsley J. (2011). Nature’s Building Blocks: An AZ Guide to the Elements.

[26] Avila D.S., Puntel R.L., Aschner M. (2013). Manganese in health and disease. Interrelat. Between Essent. Met. Ions Hum. Dis..

[27] Liu Y., Byrne P., Wang H., Koltick D., Zheng W., Nie L.H. (2014). A compact DD neutron generator–based NAA system to quantify manganese (Mn) in bone in vivo. Physiol. Meas..

[28] Subramanian K.S., Meranger J.C. (1985). Graphite furnace atomic absorption spectrometry with nitric acid deproteinization for determination of manganese in human plasma. Anal. Chem..

[29] Krebs N., Langkammer C., Goessler W., Ropele S., Fazekas F., Yen K., Scheurer E. (2014). Assessment of trace elements in human brain using inductively coupled plasma mass spectrometry. J. Trace Elem. Med. Biol..

[30] Schmitt C., Strazielle N., Richaud P., Bouron A., Ghersi-Egea J.F. (2011). Active transport at the blood-CSF barrier contributes to manganese influx into the brain. J. Neurochem..

[31] Thyssen J.P., Linneberg A., Menné T., Johansen J.D. (2007). The epidemiology of contact allergy in the general population–prevalence and main findings. Contact Dermat..

[32] Agnew U.M., Slesinger T.L. (2021). Zinc toxicity. Statpearls [Internet].

[33] Rizvi A., Parveen S., Khan S., Naseem I. (2020). Nickel toxicology with reference to male molecular reproductive physiology. Reprod. Biol..

[34] Barceloux D.G., Barceloux D. (1999). Nickel. J. Toxicol. Clin. Toxicol..

[35] Program N.T. (1996). NTP toxicology and carcinogenesis studies of nickel subsulfide (CAS No. 12035-72-2) in F344 rats and B6C3F1 mice (inhalation studies). Natl. Toxicol. Program Tech. Rep. Ser..

[36] Real M.I.H., Azam H.M., Majed N. (2017). Consumption of heavy metal contaminated foods and associated risks in Bangladesh. Environ. Monit. Assess..

[37] Rai P.K., Lee S.S., Zhang M., Tsang Y.F., Kim K.-H. (2019). Heavy metals in food crops: Health risks, fate, mechanisms, and management. Environ. Int..

[38] Alidadi H., Sany S.B.T., Oftadeh B.Z.G., Mohamad T., Shamszade H., Fakhari M. (2019). Health risk assessments of arsenic and toxic heavy metal exposure in drinking water in northeast Iran. Environ. Health Prev. Med..

[39] Wan D., Song L., Mao X., Yang J., Jin Z., Yang H. (2019). One-century sediment records of heavy metal pollution on the southeast Mongolian Plateau: Implications for air pollution trend in China. Chemosphere.

[40] Gohain M., Deka P. (2020). Trace metals in indoor dust from a university campus in Northeast India: Implication for health risk. Environ. Monit. Assess..

[41] Megertu D.G., Bayissa L.D. (2020). Heavy metal contents of selected commercially available oil-based house paints intended for residential use in Ethiopia. Environ. Sci. Pollut. Res..

[42] Tchounwou P.B., Yedjou C.G., Patlolla A.K., Sutton D.J. (2012). Heavy metal toxicity and the environment. Mol. Clin. Environ. Toxicol..

[43] Kim H.S., Kim Y.J., Seo Y.R. (2015). An overview of carcinogenic heavy metal: Molecular toxicity mechanism and prevention. J. Cancer Prev..

[44] IARC Working Group on the Evaluation of Carcinogenic Risks to Humans (2012). Arsenic and arsenic compounds. Arsenic, Metals, Fibres and Dusts.

[45] World Health Organization (2020). IARC Monographs on the Identification of Carcinogenic Hazards to Humans. https://publications.iarc.fr/Book-And-Report-Series/Iarc-Monographs-On-The-Identification-Of-Carcinogenic-Hazards-To-Humans.

[46] Bund B. (2017). Technically avoidable heavy metal contents in cosmetic products. J. Consum. Prot. Food Saf..

[47] Kessler R. (2014). Lead-Based Decorative Paints: Where are They Still Sold—And Why?. https://www.ncbi.nlm.nih.gov/pmc/articles/PMC3983718/pdf/ehp.122-A96.

[48] Apanpa-Qasim A.F., Adeyi A.A., Mudliar S.N., Raghunathan K., Thawale P. (2016). Examination of lead and cadmium in water-based paints marketed in Nigeria. J. Health Pollut..

[49] Yang F., Massey I.Y. (2019). Exposure routes and health effects of heavy metals on children. Biometals.

[50] Kim S., Eom S., Kim H.-J., Lee J.J., Choi G., Choi S., Kim S., Kim S.Y., Cho G., Kim Y.D. (2018). Association between maternal exposure to major phthalates, heavy metals, and persistent organic pollutants, and the neurodevelopmental performances of their children at 1 to 2 years of age-CHECK cohort study. Sci. Total Environ..

[51] Substances A.f.T., Registry D. (2007). CERCLA priority list of hazardous substances. https://www.atsdr.cdc.gov/spl/index.

[52] Silva F.L., Duarte T.A., Melo L.S., Ribeiro L.P., Gouveia S.T., Lopes G.S., Matos W.O. (2016). Development of a wet digestion method for paints for the determination of metals and metalloids using inductively coupled plasma optical emission spectrometry. Talanta.

[53] Izzo F.C., Balliana E., Pinton F., Zendri E. (2014). A preliminary study of the composition of commercial oil, acrylic and vinyl paints and their behaviour after accelerated ageing conditions. Conserv. Sci. Cult. Herit..

[54] Brady P. (2018). Rethinking Acrylic: Radical Solutions For Exploiting The World’s Most Versatile Medium.

[55] Nelms S. (2012). Multi-element determination in pharmaceutical preparations using the Thermo Scientific iCAP Q ICP-MS. Mercury.

[56] Wills J., Kutscher D. (2016). Analysis of pharmaceutical products for their elemental impurities with the Thermo Scientific iCAP RQ ICP-MS. Power (W).

[57] Hutton M. (1987). Human health concerns of lead, mercury, cadmium and arsenic. Leadmercurycadmium Arsen. Environ..

[58] Njati S.Y., Maguta M.M. (2019). Lead-based paints and children’s PVC toys are potential sources of domestic lead poisoning–A review. Environ. Pollut..

[59] Lidsky T.I., Schneider J.S. (2003). Lead neurotoxicity in children: Basic mechanisms and clinical correlates. Brain.

